# Now and then: a ten-year comparison of young people in residential substance use disorder treatment receiving group dialectical behaviour therapy

**DOI:** 10.1186/s12888-021-03372-2

**Published:** 2021-07-20

**Authors:** Ely M. Marceau, Gabriella Holmes, Jane Cutts, Lauren Mullaney, Denise Meuldijk, Michelle L. Townsend, Brin F. S. Grenyer

**Affiliations:** 1 School of Psychology and Illawarra Health and Medical Research Institute, University of Wollongong, Northfields Ave, Wollongong, NSW 2522 Australia; 2 Mission Australia: Triple Care Farm, 188 Knights Hill Rd, NSW 2577 Knights Hill, Australia; 3 Black Dog Institute, Faculty of Medicine, University of New South Wales, NSW 2052 Sydney, Australia

**Keywords:** Dialectical behavior therapy, Substance-related disorders, Adolescent, Residential treatment, Cohort studies

## Abstract

**Background:**

Reducing substance use in youth is a global health priority. We compared two cohorts from the same 12-week residential substance use disorder (SUD) facility over a 10 year period: Cohort A (2008–2009) and Cohort B (2018–2020). The essential components of the program remained the same with the primary treatment being dialectical behaviour therapy (DBT) plus residential milieu.

**Methods:**

Young people in the current Cohort B (*N* = 100) versus historical Cohort A (*N* = 102) had a similar ratio of males (74 vs. 70%) but were slightly older (mean 20.6 vs. 19.5 years). Linear mixed models were used to model outcome measures (global psychiatric symptoms, substance use severity, and quality of life) longitudinally up to 12 months later.

**Results:**

Baseline to end-of-treatment comparisons showed that the current Cohort B had overall higher levels of global psychiatric symptoms (*d* = 0.70), but both groups reduced psychiatric symptoms (Cohort A: *d* = 1.05; Cohort B: *d* = 0.61), and had comparable increases in confidence to resist substance use (*d* = 0.95). Longitudinal data from the current Cohort B showed significant decreases in substance use severity from baseline to 6-month follow-up (*d* = 1.83), which were sustained at 12-month follow-up (*d* = 0.94), and increases in quality of life from baseline to end-of-treatment (*d* = 0.83).

**Conclusions:**

We demonstrate how DBT plus milieu residential care for young people continues to show positive effects in a 10-year comparison. However, youth seeking treatment today compared to 10 years ago evidenced higher acuity of psychiatric symptoms reinforcing the importance of continuous improvement of psychological treatments.

**Trial registration:**

Australian New Zealand Clinical Trials Registry: trial IDACTRN12618000866202, retrospectively registered on 22/05/2018, .

**Supplementary Information:**

The online version contains supplementary material available at 10.1186/s12888-021-03372-2.

## Introduction

Adolescence is a key developmental period in which neuromaturation influences changes across biological, psychological, and social spheres of the young person [1]. Heightened risk-taking and reward seeking in this time may confer increased likelihood of young people initiating and developing problematic substance use [2–4]. Worldwide, young peoples’ use of alcohol and illicit drugs contributes significantly to overall disease burden and is now a recognised global health priority [5], highlighting the importance of effective early intervention [6]. In 2015, for young people in Australia aged 15–24, alcohol and use of illicit drugs were the leading causes of total burden of disease in males, and the second and third leading causes for females, respectively [7]. An estimated 38% of clients seeking treatment for substance use disorder (SUD) are aged under 30 [7]. Residential treatment services represent a higher-intensity level of care for SUD and may be particularly useful for clients who experience problematic substance use with comorbid mental disorders, physical health issues, or other psychosocial complexities. A small number of studies support the effectiveness of residential treatment for SUD in adult populations [8], but less is known about residential programs that cater to young people with SUD [9]. Psychosocial interventions such as family-based therapies, cognitive behavioural therapy, motivational interviewing/motivational enhancement therapy, and third-wave cognitive behavioural therapies are effective for the treatment of young people with SUD [10]. These broad psychosocial interventions may be particularly beneficial when integrated in residential treatment for young people with SUD because a range of co-occuring mental disorders are very common in this population and difficult to treat, though integrated care represents the most effective form of treatment [11, 12].

Dialectical behaviour therapy (DBT) was developed by Marsha Linehan [13] as an adaptation of cognitive behavioural therapy for the treatment of chronic self-harm and suicidality. It is an evidence-based psychotherapy for the treatment of borderline personality disorder (BPD) [14] with demonstrated effects on neural activation [15], and has also been adapted for the treatment of adolescents and young people with BPD symptoms [16]. Additional research studies have demonstrated the effectiveness of DBT as a treatment for co-occurring SUD (e.g. [17–19]). Despite this, to the best of our knowledge, there are no studies that have evaluated integrated residential treatment for young people with SUD where DBT-based interventions form a major component of treatment. This is exemplified by the results of a recent systematic review of residential SUD treatment in which only three of the included 23 studies involved a specific focus on treating the young adult age group (i.e., 18–24 years) [8]. These studies found improved outcomes for young people in integrated programs incorporating the following psychosocial interventions: cognitive behavioural and motivational enhancement approaches [20]; unspecified individual and group evidence-based interventions based on an integrated dual diagnosis model [21]; and family-based, cognitive behavioural, and motivational enhancement approaches [22].

Given the lack of studies evaluating residential SUD treatment for young people and the strong need for integrated, evidence-based approaches to improve outcomes for this vulnerable group, the current study used two waves of data from a residential SUD facility for young people, in which DBT forms a major component of treatment. Specifically, we aimed to evaluate treatment outcomes in a pragmatic trial with available data from two cohorts 10-years apart (2008–2009 and 2018–2020), hypothesising that both groups would demonstrate improvements in outcomes following treatment. Specifically, designated outcomes were global psychiatric symptoms (primary outcome), as well as substance use severity and quality of life (secondary outcomes). The primary outcome was chosen on the basis of the high co-occurrence of SUD and mental disorders in adolescents [11, 12], the relevance of mental health outcomes in studies of residential SUD programs [8], and the context of the residential facility studied, which is focused on providing integrated mental health treatment and a holistic approach for residents that often present with comorbid mental health and psychosocial challenges. The pragmatic design of this trial additionally allowed changes in presentation over time to be investigated, and we subsequently used the opportunity to investigate whether differences emerged in sociodemographics or psychiatric symptom severity in the young people accessing this service over a 10-year period.

## Methods

### Participants

Participants were residents of Triple Care Farm (TCF), a residential rehabilitation and treatment program for young people aged 16–24 years located in the NSW Southern Highlands, Australia. Data for Cohort A were routinely collected and archived, being made available for this study in de-identified form following ethical approval. Cohort B were recruited from TCF between 2018 and 2020. All participants met diagnostic criteria for at least one SUD according to DSM-IV criteria, following their admission to a high-intensity level of treatment (i.e., residential program). Inclusion criteria were as follows: (i) SUD on referral; (ii) age 16–24 years; (iii) completion of detoxification prior to treatment entry for the residential rehabilitation program; (iv) fluency in English to a level ensuring comprehension of study requirements. Though there are no routine tests of abstinence at TCF, young people sign a code of conduct and agreement (n.b., as TCF is a harm reduction program, a young person’s long-term goal may not be abstinence but reduction of use or associated harms).

The study was registered as a clinical trial (Australian New Zealand Clinical Trials Registry: ANZCTR; trial ID ACTRN12618000866202, date registered 22/05/2018), with approval granted by the University of Wollongong Health and Medical Human Research Ethics Committee (reference 2017/233) and the Aboriginal Health and Medical Research Council (AH&MRC; reference 1319/17). During recruitment of Cohort B, adult participants (i.e., those aged 18 years and older) provided written informed consent following a full explanation of study procedures (n.b., for participants under 18 years of age, legal guardians provided consent, while these participants provided assent to participate in the study).

### Clinical and social assessments

Basic sociodemographic and substance use data were gathered through a semi-structured interview following a routine intake assessment. Subscales of the Brief Symptom Inventory [23] provided a snapshot of psychiatric symptom domains at baseline. Cohort A had the same primary measures as Cohort B and were assessed at baseline, 6-weeks (mid-treatment), and 12-weeks (end-of-treatment), but as shown in Tables 1 and 3 some measures (e.g. Severity of Dependence Scale, Quality of Life) were missing for Cohort A. Thus some analyses focused on Cohort B only (e.g. Table 3).

### Measures

#### Primary outcome measure: global psychiatric symptoms

Given the high co-occurrence of SUD and mental disorders, as well as the relevance of mental health outcomes in studies of residential SUD programs [8], the Brief Symptom Inventory (BSI) [23] Global Severity Index was the primary outcome measure and was used to assess level of global psychiatric symptoms. The BSI is 53-item self-report questionnaire consisting of nine subscales (Somatisation, Obsessive Compulsive, Interpersonal Sensitivity, Depression, Anxiety, Hostility, Phobic Anxiety, Paranoid Ideation, and Psychoticism) that yield three global indices (Global Severity Index, Positive Symptoms Distress Index, Positive Symptoms Total). Participants were instructed to indicate how much each item has distressed or bothered them during the past 7 days including today (5-point Likert scale from 0 “not at all” to 4 “extremely”). Example items include: “Feeling tense or keyed up”, “Feeling nervous when you are left alone”, and “Feelings of guilt”.

#### Secondary outcome measures

##### Severity of Dependence Scale (SDS)

The SDS is a 5-item self-report questionnaire used to assess severity of dependence [24]. Items (e.g., “Do you think your use of (substance) was out of control?”) are rated on a 4-point Likert scale, in relation to the self-identified primary drug of concern.

##### Brief Situational Confidence Questionnaire (BSCQ)

The BSCQ is an 8-item self-report questionnaire in which participants rate their percentage of confidence from 0 to 100 in resisting precipitants to relapse, including social pressure to drink, unpleasant emotions, and urges and temptations [25].

##### World Health Organisation Quality of Life-8 (QOL)

Quality of life was measured using 8 items from the World Health Organisation Quality of Life Short (QOL) [26].

#### Treatment satisfaction and integrity measures

##### Group Session Rating Scale (GSRS)

The GSRS is a 4-item self-report questionnaire that measures group therapy alliance [27]. Items evaluate the relationship, goals and topics, acceptability of approach, and a sense of overall fit in the group therapy alliance and are presented on a 10 cm line with bipolar anchors (e.g., “‘I felt understood, respected, and accepted by the leader and the group” to “I did not feel understood, respected, and/or accepted by the leader and/or the group”).

##### Treatment Integrity Checklist (TIC)

The TIC was developed to measure fidelity to the treatment manual. Each session was rated on seven objectives including orientation and introductions, here and now interpersonal focus, mindfulness activity, practice review, short break, skills training, and mindfulness activity/summary time. The objectives were rated as ‘not in place,’ ‘partially in place,’ or ‘completely in place’ by the co-facilitator of the session. Similar treatment integrity checklists for DBT have been used in previous research [28].

### Design and procedures

**Fig. 1:**
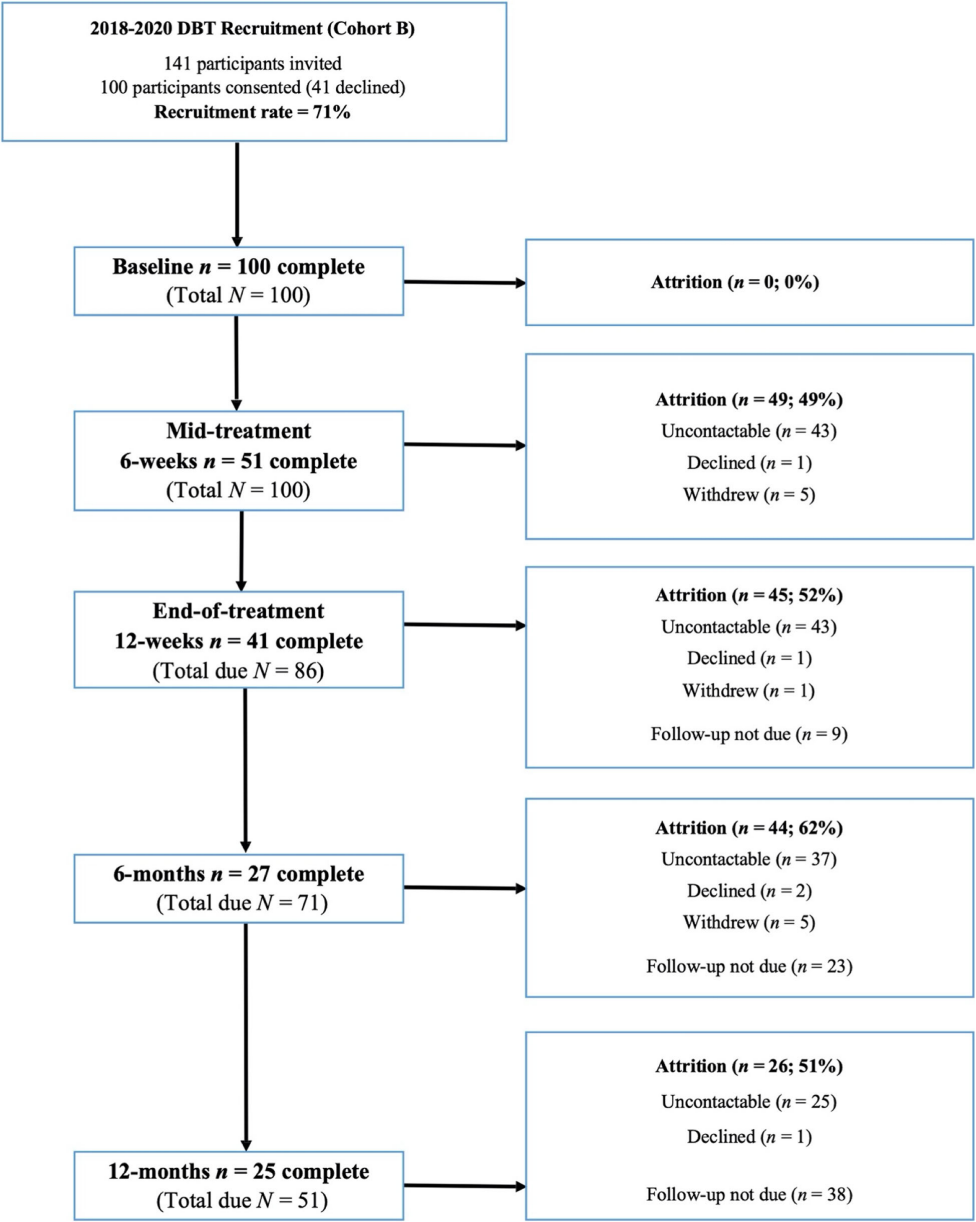
Participant flowchart detailing recruitment rate, number of participants assessed, attrition rates, and status of participants who were unable to be assessed at each timepoint (2018–2020; Cohort B)

Measures for Cohort A were collected at the time and then archived, being made available for this study following ethical approval. For Cohort B, participants initially completed a face-to-face baseline assessment session with a research assistant. Follow-up assessments were subsequently completed face-to-face (for participants still in treatment) or over the phone (for participants who had completed or dropped out of treatment). There were up to 5 contact attempts for participants at each timepoint (baseline, 6-weeks, 12-weeks, 6-months, 12-months) and the following incentives were used: Participants received reimbursement via gift cards at 12-weeks and 6-months ($20), and at 12-months ($50). A participant flowchart is illustrated in Fig. 1, including attrition rates at each timepoint. Data collection for Cohort B occurred from January 2018 to March 2020. Due to resource constraints in the current study, a subsample of participants in the DBT group were followed up at each timepoint. These were drawn from only those participants with follow-up assessments due during the funding period (see “total due” in Fig. 1). Rates of attrition are thus expressed as a percentage of total follow-up assessments due at this point in time.

#### Residential milieu therapy

The residential setting remained the same over the 10 year study period, and is an 18-bed program for young people aged between 16 and 24 years, which incorporates the general principles of milieu treatment [29]. The treatment model is a 12-week holistic psychosocial rehabilitation program based on harm minimisation and health promotion. This 12-week program is followed up with community aftercare. The DBT treatment program is embedded within the overall residential milieu. Within the milieu there were general worker-resident activities throughout the day and evening designed to provide therapeutic containment and safety whilst seeking to generalise the use of skills learnt in the DBT program. The wider residential milieu also included principles from psychoeducation, motivational interviewing for substance use and criminality, relapse prevention, strengths-based work, and drug education.

#### Dialectical behaviour therapy (DBT) intervention

The DBT intervention was based on manualised DBT treatment [30] that was adapted for the Australian context of working with young people in a residential SUD setting. Existing DBT principles were tailored to working with young people [31] in the residential treatment setting [32] over a shorter time frame [33], with a primary treatment target of SUD [34]. The DBT-based intervention at TCF also draws upon the Modular Practice Elements Approach, which recognises that therapeutic interventions are comprised of numerous discrete and separable elements [35, 36]. Treatment providers were trained and treatment integrity was monitored by co-facilitators during treatment of Cohort B. All resources to facilitate the group are available online [37, 38], as well as an accompanying website [39].

The group format at TCF is an ‘open’ group, as the program has a rolling intake, with new young people entering the program and joining groups each week. In order to ensure that group cohesion is maintained throughout this process, new young people are formally oriented through a pre-treatment session which occurs prior to commencing group. This introduction familiarises participants with the structure, expectations, overarching goals, and principles of the approach. Following the pre-treatment session, young people commence group sessions, with 2 × 2-h sessions occurring each week of the program, facilitated by a psychologist and co-facilitated by another psychologist or residential staff member.

### Statistical methods

All statistical analyses were performed using IBM Statistical Package for the Social Sciences (SPSS version 25) [40]). During data cleaning, a number of missing values were detected for Cohort B though Little’s [41] MCAR test indicated that there was not sufficient evidence to conclude that data were not missing completely at random (all *p*s > .221).

Initially, descriptive statistics were used to explore the clinical and demographic characteristics of both groups at baseline, client treatment satisfaction scores, and treatment integrity. To further understand the impact of attrition, baseline characteristics for subgroups of Cohort B group were compared (i.e., those lost to 12-month follow-up vs. 12-month follow-up completers). A series of linear mixed models were then used to compare Cohorts A and B on outcome measures over time (BSI and BSCQ; utilising the first three timepoints due to availability of Cohort A data). Linear mixed models were also used to investigate changes over time in outcomes of Cohort B only (SDS and QOL; utilising all five timepoints). Intention-to-treat analyses were used and the models included fixed effects for group (for group comparisons), time, and the group x time interaction (where applicable), and random effects for participants. All linear mixed models utilised Sidak adjustment for multiple comparisons where pairwise comparisons or simple effect analyses were required. Additionally, time was treated categorically in all analyses and thus only random intercept models were considered. A top-down model building strategy was utilised and in all analyses variance components structures for the random effects and residuals provided the best model fit [42]. Detailed methods for calculation of repeated-measures effect sizes [43, 44] are provided as supplementary material (see Additional file 1).

## Results

### Demographic and baseline characteristics

Table 1 illustrates baseline characteristics of Cohort B (2018–2020) and historical Cohort A (2008–2009) groups. Cohort B group were older (*p* < .001, *d* = 0.52) and had completed more years of school education (*p* < .001, *d* = 0.69) compared to Cohort A. Overall BSI scores were higher for those in Cohort B compared to their historical counterparts (*p* = .02, *d* = 0.36). Exploratory analyses (n.b., uncorrected for multiple comparisons) indicated Cohort B (vs. Cohort A) endorsed higher scores on a number of scales; Positive Symptoms Total (*p* = .027, *d* = 0.34), Obsessive Compulsive (*p* = .044, *d* = 0.31), Interpersonal Sensitivity (*p* = .002, *d* = 0.47), Anxiety (*p* = .01, *d* = 0.39), Phobic Anxiety (*p* < .001, *d* = 0.58), Paranoid Ideation (*p* = .022, *d* = 0.35), and Psychoticism (*p* = .002, *d* = 0.48).

**Table 1:** Baseline characteristics of young people in residential substance use disorder treatment receiving dialectical behaviour therapy (DBT): Cohort A (2008–2009) versus Cohort B (2018–2020) groups

Characteristic	Cohort B (***n*** = 100)	Cohort A (***n*** = 102)	***p***	***d***
Age (*M*, SD)	20.6 (2.0)	19.5 (2.2)	<.001***	0.52
Gender (*n*, % male)	74 (74.0)	71 (69.6)	.49	
Education (completed school years; *M*, SD)	10.5 (1.1)	9.7 (1.2)	<.001***	0.69
Completed further study (*n*, %)	19 (19.0)			
Technical/Trade Certificate	18 (18.0)			
Bachelor-level College	1 (1.0)			
Employment status (*n*, %)				
Unemployed	66 (66.0)			
Full- or part-time employment	26 (26.0)			
Receiving pension or allowance	8 (8.0)			
Accommodation (*n*, %)				
(Residing with) rental tenant/home owner	80 (80.0)			
Homeless	13 (13.0)			
Inpatient, forensic setting, hostel, other	7 (7.0)			
Relationship status (*n*, % single)	78 (78.0)			
Primary problematic substance (*n*, %)^a^				
Cannabis	38 (38.8)			
Amphetamine-type stimulants	31 (31.6)			
Alcohol	15 (15.3)			
Cocaine	4 (4.1)			
Sedatives	4 (4.1)			
Heroin	3 (3.1)			
Hallucinogens	1 (1.0)			
Steroids	1 (1.0)			
Nicotine	1 (1.0)			
Injected during last 3 months (*n*, %)^b^	5 (6.8)			
Overdosed (any drug) last 3 months (*n*, %)^b^	21 (28.4)			
Arrested in the last 3 months (*n*, %)^b^	13 (17.6)			
Brief Symptom Inventory (*M*, SD)^c^				
Global Severity Index	67.9 (11.9)	63.7 (11.7)	.020*	0.36
Positive Symptoms Distress Index	64.2 (9.3)	61.6 (10.4)	.09	
Positive Symptoms Total	65.4 (9.9)	61.8 (11.5)	.027*	0.34
Somatisation	62.2 (11.7)	59.3 (12.2)	.11	
Obsessive Compulsive	68.3 (10.0)	65.1 (10.5)	.044*	0.31
Interpersonal Sensitivity	62.8 (13.0)	56.7 (12.9)	.002**	0.47
Depression	65.9 (11.3)	62.6 (11.6)	.06	
Anxiety	66.4 (11.7)	61.7 (12.3)	.010*	0.39
Hostility	59.9 (11.1)	58.6 (12.3)	.49	
Phobic Anxiety	63.8 (10.7)	57.2 (12.1)	<.001***	0.58
Paranoid Ideation	63.4 (11.8)	59.2 (12.1)	.022*	0.35
Psychoticism	69.1 (10.7)	63.7 (11.9)	.002**	0.48
Brief Situational Confidence Questionnaire (*M*, SD)^d^	45.02 (24.8)	45.8 (23.5)	.832	

^a^*n* = 98

^b^*n* = 74

^c^*n* = 78 for DBT group only

^d^*n* = 77 for DBT group only

* *p* < .05. ** *p* < .01. *** *p* < .001

Analyses of days between each follow-up timepoint for Cohort B were also conducted: Baseline to 6-week follow-up: *M* = 46.4, *SD* = 6.9, range = 39–67; Baseline to 12-week follow-up: *M* = 88.7, *SD* = 13.0, range = 63–133; Baseline to 6-month follow-up: *M* = 205.6, *SD* = 24.3, range = 177–277; Baseline to 12-month follow-up: *M* = 378.3, *SD* = 23.8, range = 328–446.

To investigate the role of attrition, a subgroup analysis comparing baseline characteristics of those in the Cohort B who were lost to 12-month follow-up (*n* = 76) to 12-month follow-up completers (*n* = 24) was conducted and is presented in Table 2. Utilising all available baseline variables including demographics and scores on primary and secondary outcomes, the only significant differences that emerged indicated that those lost to 12-month follow-up reported less symptoms on the Somatisation scale of the BSI (*p* = .022, *d* = 0.65) and higher quality of life (*p* = .007, *d* = 0.62) at baseline than their counterparts who completed 12-month follow-up.

**Table 2:** Subgroup analysis of baseline characteristics of young people in residential substance use disorder treatment (Cohort B) 2018–2020: Lost to 12-month follow-up vs. 12-month follow-up completers

Characteristic	Lost to 12-month follow-up	Completed 12-month follow-up	***p***	***d***
Age (*M*, SD)^a^	20.7 (2.0)	20.5 (1.8)	.781	
Gender (*n*, % male)^a^	55 (72.4)	19 (79.2)	.508	
Education (completed school years; *M*, SD)^a^	10.6 (1.0)	10.2 (1.4)	.117	
Completed further study (*n*, %)#			1.0	
Technical/Trade Certificate	14 (93.3)	4 (100.0)		
Bachelor-level College	1 (6.7)	0 (0)		
Employment status (*n*, %)^a^			.374	
Unemployed	49 (64.5)	17 (70.8)		
Full- or part-time employment	22 (28.9)	4 (16.7)		
Receiving pension or allowance	5 (6.6)	3 (12.5)		
Accommodation (*n*, %)^a^			.777	
(Residing with) rental tenant/home owner	62 (81.6)	18 (75.0)		
Homeless	9 (11.8)	4 (16.7)		
Inpatient, forensic setting, hostel, other	5 (6.6)	2 (8.3)		
Relationship status (*n*, % single)^a^	60 (78.9)	18 (75.0)	.684	
Primary problematic substance (*n*, %)^b^			.787	
Cannabis	26 (34.7)	12 (52.2)		
Amphetamine-type stimulants	26 (34.7)	5 (21.7)		
Alcohol	11 (14.7)	4 (17.4)		
Cocaine	4 (5.3)	0		
Sedatives	3 (4.0)	1 (4.3)		
Heroin	2 (2.7)	1 (4.3)		
Hallucinogens	1 (1.3)	0		
Steroids	1 (1.3)	0		
Nicotine	1 (1.3)	0		
Injected during last 3 months (*n*, %)^c^#	4 (7.0)	1 (5.9)	1.0	
Overdosed (any drug) last 3 months (*n*, %)^c^#	15 (26.3)	6 (35.3)	.544	
Arrested in the last 3 months (*n*, %)^c^#	10 (17.5)	3 (17.6)	1.0	
Brief Symptom Inventory (*M*, SD)^d^				
Global Severity Index	66.7 (12.4)	71.4 (9.7)	.125	
Positive Symptoms Distress Index	63.0 (8.8)	67.5 (10.2)	.059	
Positive Symptoms Total	64.3 (10.1)	68.3 (8.8)	.118	
Somatisation	60.4 (12.1)	67.3 (9.0)	.022*	0.65
Obsessive Compulsive	67.1 (10.2)	71.6 (8.7)	.083	
Interpersonal Sensitivity	61.4 (12.8)	66.8 (13.1)	.110	
Depression	65.0 (11.1)	68.6 (11.6)	.216	
Anxiety	65.3 (12.3)	69.6 (9.5)	.161	
Hostility	59.2 (10.6)	61.6 (12.6)	.416	
Phobic Anxiety	62.6 (10.7)	67.4 (9.9)	.081	
Paranoid Ideation	62.6 (12.0)	65.7 (11.1)	.310	
Psychoticism	68.3 (10.9)	71.4 (10.1)	.270	
Brief Situational Confidence Questionnaire (*M*, SD)^e^	45.9 (25.2)	42.2 (24.1)	.573	
Severity of Dependence Scale (*M*, SD)^f^	9.1 (3.1)	10.4 (2.7)	.127	
World Health Organisation Quality of Life-8 (*M*, SD)^g^	3.3 (0.7)	2.8 (0.9)	.007**	0.62

^a^lost *n* = 76; completed *n* = 24

^b^lost *n* = 75; completed *n* = 23

^c^lost *n* = 57; completed *n* = 17

^d^lost *n* = 58; completed *n* = 20

^e^lost *n* = 58; completed *n* = 19

^f^lost *n* = 57; completed *n* = 19

^g^lost *n* = 60; completed *n* = 19

# = fisher’s exact test

* *p* < .05. ** *p* < .01. *** *p* < .001

### Primary outcome: psychiatric symptoms

Table 3 depicts observed scores of primary and secondary outcomes. Brief Symptom Inventory Global Severity Index (GSI) linear mixed model results indicated significant effects of time, *F* (4, 221) = 12.54, *p* < .001, group, *F* (1, 236) = 20.08, *p* < .001, and the time x group interaction, *F* (2, 224) = 4.17, *p* = .017. The current Cohort B group had overall higher BSI scores compared to the historical Cohort A group (*M* difference = 8.3, SE = 1.8, *p* < .001, *d* = 0.70). Significant decreases in GSI scores were observed for both groups from baseline to end-of-treatment (Cohort B: *M* difference = − 7.3, *p* = .003, *d* = 0.61; Cohort A: *M* difference = − 11.7, *p* < .001, *d* = 1.05) and Cohort A also displayed a significant decrease from baseline to mid-treatment (*M* difference = − 8.0, *p* < .001), while Cohort B did not (*M* difference = − 1.86, *p* = .961), representing a between-groups effect size of *d* = − 0.83.

**Table 3:** Observed scores for Cohort B (2018–2020) and Cohort A (2008–2009) groups of young people in residential substance use disorder treatment receiving dialectical behaviour therapy: Psychiatric symptoms, substance use, and quality of life outcomes

Outcome	Cohort B		Cohort A	
	***n***	***M*** (SD)	***n***	***M*** (SD)
Psychiatric symptoms				
BSI				
Baseline	78	67.9 (11.9)	102	63.7 (11.7)
6-weeks (mid-treatment)	40	67.4 (10.1)	62	55.2 (14.0)
12-weeks (end-of-treatment)	28	62.6 (12.4)	21	50.9 (13.5)
6-months	27	67.8 (11.9)		
12-months	24	68.6 (11.5)		
Substance use				
BSCQ				
Baseline	77	45.0 (24.8)	102	45.8 (23.5)
6-weeks (mid-treatment)	40	67.0 (22.7)	63	67.3 (20.2)
12-weeks (end-of-treatment)	28	65.0 (29.5)	7	74.4 (22.3)
6-months	27	50.1 (28.6)		
12-months	24	54.3 (31.6)		
SDS				
Baseline	76	9.5 (3.0)		
6-weeks (mid-treatment)	40	7.7 (3.3)		
12-weeks (end-of-treatment)	28	8.6 (4.0)		
6-months	27	5.0 (3.5)		
12-months	24	5.7 (5.0)		
Quality of life				
QOL				
Baseline	79	3.2 (0.8)		
6-weeks (mid-treatment)	40	3.6 (0.6)		
12-weeks (end-of-treatment)	26	3.7 (0.7)		
6-months	27	3.2 (0.7)		
12-months	24	3.4 (0.7)		

*BSCQ* Brief Situational Confidence Questionnaire, *BSI* Brief Symptom Inventory Global Severity Index, *SDS* Severity of Dependence Scale, *QOL* World Health Organisation Quality of Life-8

### Secondary outcomes: substance use and quality of life

Linear mixed model results for Brief Situational Confidence Questionnaire (BSCQ) scores revealed a significant effect of time, *F* (4, 237) = 19.86, *p* < .001, but non-significant effects for group, *F* (1, 325) = 0.46, *p* = .496, and the time x group interaction, *F* (2, 242) = 0.11, *p* = .894. BSCQ scores increased from baseline to mid-treatment (*M* difference = 21.2, *p* < .001, *d* = 0.98), and these improvements were sustained at end-of-treatment (*M* difference = 26.5, *p* < .001, *d* = 0.95). Follow-up data indicated that improvements were not maintained as there was a decrease in BSCQ scores from mid-treatment to 6-month follow-up (*M* difference = − 14.8, *p* = .021, *d* = − 0.62) and end-of-treatment to 6-month follow-up (*M* difference = − 20.1, *p* = .018, *d* = − 0.62).

**Fig. 2:**
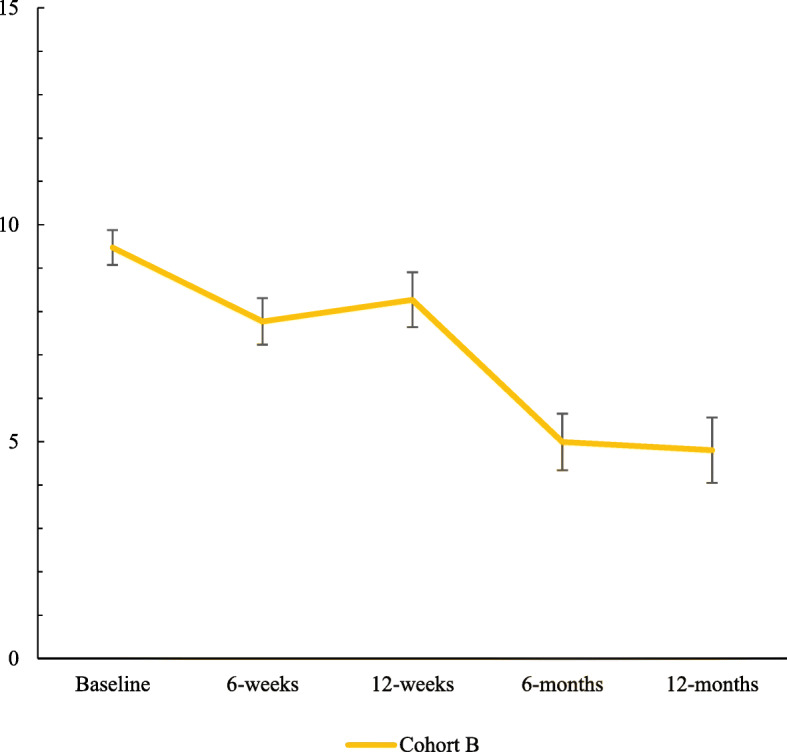
Changes in Severity of Dependence Scale (SDS) scores over time for young people in residential substance use disorder treatment receiving dialectical behaviour therapy (DBT: 2018–2020; Cohort B)

Severity of Dependence Scale (SDS) scores in the Cohort B revealed a significant effect of time, *F* (4, 142) = 15.39, *p* < .001 and are illustrated in Fig. 2. Significant decreases in SDS scores occurred from baseline to 6-month follow-up (*M* difference = − 4.6, *p* < .001, *d* = 1.83), and these improvements were sustained at 12-month follow-up (*M* difference = − 4.2, *p* < .001, *d* = 0.94). Significant decreases also occurred from mid-treatment to 6-month follow-up (*M* difference = − 2.9, *p* = .002, *d* = 0.87) and 12-month follow-up (*M* difference = − 2.5 points, *p* = .014, *d* = 0.58), and end-of treatment to 6-month follow-up (*M* difference = − 3.3, *p* = .001, *d* = 0.66) and 12-month follow-up (*M* difference = − 3.0, *p* = .006, *d* = 0.06).

World Health Organisation Quality of Life-8 (QOL) scores in Cohort B revealed a significant effect of time, *F* (4, 133) = 5.80, *p* < .001. Significant increases in QOL scores occurred from baseline to mid-treatment (*M* difference = 0.4, *p* = .034, *d* = 0.44) and these improvements were sustained at end-of-treatment (*M* difference = 0.6, *p* < .001, *d* = 0.83).

### Treatment integrity and satisfaction

During the course of the DBT intervention for Cohort B, 31 out of 60 (52%) introductory DBT group sessions and 154 out of 384 (40%) ongoing DBT group sessions were rated for treatment integrity by the co-facilitator using the Treatment Integrity Checklist. Strong fidelity to the manual was demonstrated by a high percentage of ‘completely in place’ ratings across all areas assessed (72.1 and 83.5% for introductory and group sessions, respectively). Client treatment satisfaction was measured by responses on the Group Session Rating Scale. There were 1242 individual responses that demonstrated a high level of client satisfaction across all domains: Relationship *M* = 8.9, Mdn = 9.5, SD = 1.6, range = 0.4–9.6; Goals and Topics *M* = 8.7, Mdn = 9.5, SD = 1.7, range = 0–10; Approach/Method *M* = 8.8, Mdn = 9.5, SD = 1.6, range = 0–10; Overall *M* = 8.9, Mdn = 9.5, SD = 1.6, range = 0–10.

## Discussion

Using a cohort-based design, we evaluated DBT as an intervention component for young people with SUD in the context of an integrated residential SUD treatment program, using comparative data over a 10-year period. We found that both the contemporary Cohort B group (2018–2020) and historical Cohort A group (2008–2009) improved on primary and secondary outcome measures during a 12-week residential program. Although at baseline Cohort B displayed overall higher levels of global psychiatric symptoms (*d* = 0.70), both groups demonstrated reductions in psychiatric symptoms over time. Improvements were larger for Cohort A compared to their contemporary counterparts (*d* = 1.05 vs. *d* = 0.61) and occurred earlier during the residential program (i.e., Cohort A vs. Cohort B mid-treatment *d* = 0.83). With regard to substance use outcomes, increased confidence in resisting the urge to use substances was similar for both groups. Improvements were evident at mid-treatment (*d* = 0.98) and were sustained at end-of-treatment (*d* = 0.95). At 6-month follow-up, however, these improvements had declined (*d* = − 0.62). Substance use severity was assessed within the Cohort B only and showed large decreases evident from baseline to 6-month follow-up (*d* = 1.83), which were sustained at 12-month follow-up (*d* = 0.94). Cohort B also demonstrated increased quality of life from baseline to mid-treatment (*d* = 0.44) and these improvements were sustained at end-of-treatment (*d* = 0.83). Treatment integrity and client treatment satisfaction for Cohort B were both highly rated by co-facilitators and participants, respectively.

These findings are congruent with numerous studies indicating that DBT leads to improved clinical outcomes across diagnostic groups and clinical settings [14, 16, 18, 45], but to our knowledge is the first to show consistency of effects over a decade, and evaluate DBT within the context of an integrated residential SUD treatment program [8]. DBT has been conceptualised as a *transdiagnostic psychotherapy* [46] due to its similar positive impacts across diverse settings and diagnostic groups, including people with SUD. These results lend further evidence to support this notion. Though DBT was associated with improved outcomes for both Cohort A and B, an unexpected finding in the present study concerned differences in the profiles of young people accessing residential SUD treatment over the past decade. Of particular note were the higher levels of psychiatric symptoms found in Cohort B (vs. their 2008–2009 counterparts) reflected by both global severity of symptoms and in specific symptom domains. Cohort B were also both older and had more years of school education than Cohort A. One contextual factor that may have influenced these findings relates to changes in school leaving age that were introduced in 2009 for New South Wales, Australia (i.e., as of 1 January 2010, the minimum school leaving age was raised from 15 to 17 years [47]). Despite these differences, both groups reported similar levels of confidence in resisting the urge to use substances. Crucially, these findings reflect that residential SUD treatment for young people may increasingly be required to accommodate clients with heightened levels of psychiatric symptoms. This finding could also be understood in the context of increased availability of community-based programs catering to young people experiencing symptoms of a mild to moderate severity level in Australia [48]. It could also reflect a true increase in numbers of young people experiencing – or seeking help for – SUD and elevated comorbid mental health symptoms. A further possibility is that these differences could be associated with changes in drug use profiles over time (n.b., primary problematic substance data was available only for Cohort B and this precluded comparisons between cohorts). Whatever the reasons for these changes over the past decade, the current study provides some evidence to support residential SUD treatment using DBT and milieu care as an effective intervention for young people experiencing SUD and comorbid psychiatric symptoms. Further to this, given these differences in the characteristics of the cohorts investigated, future studies that test potential moderators of treatment response may help to garner more knowledge regarding factors related to better (or poorer) treatment outcomes [49, 50].

There are a number of limitations in the present study. First, while the DBT intervention was associated with significant improvements in primary and secondary outcomes over time for both Cohorts A and B, the study did not include an active comparison condition. This precludes conclusions being made regarding specific elements of the intervention that resulted in improved outcomes as there are a number of non-specific elements and/or broader components of the residential milieu program that may have contributed to improved outcomes. Replication of these findings in future multi-site randomised controlled trials including well-matched active comparison conditions would allow firmer conclusions regarding the mechanisms of these effects – disentangling the purported beneficial effects of DBT (or any other specific psychological intervention) from wider whole-of-program effects. This limitation is also linked to a strength of the present study, with the inclusion of a historical comparison sample creating the possibility to observe changes in the characteristics of young people accessing residential SUD treatment over the past decade (i.e., 2008–2020). A second limitation concerns the lack of diagnostic information regarding comorbid mental disorders. Future studies should include formal assessment of psychiatric diagnoses for more sensitive reporting of clinical characteristics and comorbidities, and their effects on treatment processes. Thirdly, resource constraints meant the treatment integrity ratings of the DBT intervention for Cohort B were made by co-facilitators rather than independent observers. A notable strength of the present study was the inclusion of longitudinal follow-up over five timepoints, including 6- and 12-months post-treatment for Cohort B, contributing to understanding of longer-term trajectories of change and effects of the intervention over time. Though there were considerable attrition rates ranging from 49 to 62% in Cohort B, they are comparable to those observed in SUD treatment studies [51], with reported follow-up rates ranging from 36 to 100% [52]. All available data, however, were utilised in recognition of the challenge and value of obtaining longitudinal SUD treatment data, particularly involving young people. There is debate concerning the parameters for acceptable attrition rates and resultant effects on internal validity [53, 54] and therefore the findings in the present study warrant cautious interpretation and require replication. Though we found minimal differences in baseline characteristics when comparing 12-month follow-up completers to those who were lost to 12-month follow-up in Cohort B, it must be clearly acknowledged that these attrition rates present uncertainty regarding the stability of outcomes in this study and may introduce bias. For example, a longitudinal study of adolescent psychiatric outpatients found that presence and severity of psychological disorders at 2-year follow-up was related to degree of difficulty in making contact [55]. Notwithstanding the challenges of this kind of research, future longitudinal studies that are appropriately planned and resourced to maximise follow-up rates will ensure that findings over time are generalisable to the relevant SUD population [56]. Related to this, future studies may investigate trajectories of improvement over time, as it is noteworthy that some treatment gains in the current study were evident at the 6-week follow-up (i.e., mid-treatment). Studies investigating the effects of treatment length on outcomes may provide data to support appropriately matching treatment intensity to client need, and are congruent with a stepped care approach (e.g. [57, 58],).

## Conclusions

We are the first to report how an integrated DBT plus milieu approach to care for young people in residential SUD treatment demonstrated sustained positive treatment outcomes in two cohorts a decade apart: 2008–2009 (Cohort A) and 2018–2020 (Cohort B). With higher rates of psychiatric symptoms identified in the Cohort B, future studies investigating moderators and mechanisms of treatment may be beneficial [59], along with novel developments in intervention approaches to promote early intervention and improved outcomes for young people with drug and alcohol addiction who have increased severity and comorbidity.

## Supplementary Information

**Additional file 1.** Calculation of repeated-measures effect sizes. Methods for calculation of repeated-measures effect sizes.

## Data Availability

The datasets generated and/or analysed during the current study are not publicly available as participants did not give consent for release beyond the study. Please contact the corresponding author (EMM) for requests.
