# Supplementary material for: Now and then: a ten-year comparison of young people in residential substance use disorder treatment receiving group dialectical behaviour therapy
Source: BMC Psychiatry. 2021 Jul 20;21:362. doi: 10.1186/s12888-021-03372-2 (PMC8293584; doi:10.1186/s12888-021-03372-2)
Supplement: Supplementary file 1 — Additional file 1. Calculation of repeated-measures effect sizes. Methods for calculation of repeated-measures effect sizes. [file 12888_2021_3372_MOESM1_ESM.docx]

**Calculation of repeated-measures effect sizes**

Repeated-measures effect sizes were calculated by estimating the standardised mean difference between relevant timepoints (for Cohort A and Cohort B groups separately to examine within-group changes, and in combination to examine significant effects of time) using the following formula (Borenstein, Hedges, Higgins, & Rothstein, 2009)

$$d \text{=}\frac{\bar{Y}_{diff}}{S_{within}} ,$$

where $\bar{Y}_{diff}$ is the mean pretest – posttest scores and $S_{within}$ is the within-group standard deviation given by

$$S_{within}=\frac{S_{diff}}{\sqrt{2(1-r)}} ,$$

where $S_{diff}$ is the standard deviation of the difference scores and $r$ is the pretest-posttest correlation. For significant time x group interactions, between-group effect sizes were calculated to examine differences in magnitude of change between Cohort A and Cohort B groups. These effect sizes were calculated for linear mixed models using the equation recommended by Feingold (2009)

$$d=\frac{\left( b*duration \right)}{SD} ,$$

where $b$ is the unstandardized coefficient for a fixed effect of a binary time-invariant covariate on slope for linear trend, $duration$ is the number of timepoints minus one, and $SD$ is the pooled within-group variability at baseline given by

$$SD=\sqrt{\frac{\left( n_{1}-1 \right)S_{1}^{2}+\left( n_{2}-1 \right)S_{2}^{2}}{n_{1}+n_{2}-2}} .$$
